# Response mechanism of water status and photosynthetic characteristics of *Cotoneaster multiflorus* under drought stress and rehydrated conditions

**DOI:** 10.3389/fpls.2024.1457955

**Published:** 2025-01-14

**Authors:** Qiu-liang Huang, Miao-miao Zhang, Chang-shun Li, Bo-yang Li, Sheng-lan Zhuo, Yu-shan Yang, Yu-da Chen, An-na Zhong, Hao-yang Liu, Wen-feng Lai, Zhen-bei Huang, Ming-hui Cao, Zong-sheng Yuan, Guo-fang Zhang

**Affiliations:** ^1^ College of Forestry, Fujian Agriculture and Forestry University, Fuzhou, Fujian, China; ^2^ Service Center, Fujian Meteorological Bureau, Fuzhou, Fujian, China; ^3^ Production Technology Department, Fujian Minhou Baisha State-Owned Forest Farm, Fuzhou, Fujian, China; ^4^ Institute of Oceanography, Minjiang University, Fuzhou, Fujian, China

**Keywords:** *Cotoneaster multiflorus*, drought stress, photosynthetic characteristics, water status, nocturnal sap flow, high-throughput sequencing, high-strategy

## Abstract

**Introduction:**

Plant physiology response and adaptation to drought stress has become a hotspot in plant ecology and evolution. *Cotoneaster multiflorus* possesses high ecological, ornamental and economic benefits. It has large root system and tolerance to cold, drought and poor soil. Therefore, *C. multiflorus* is considered as one of the most important tree species for ecological restoration in arid and semi-arid areas. However, little is known about the physiological mechanisms, molecular mechanisms and drought strategies of how *C. multiflorus* responds to drought stress. Therefore, exploring the physiological response mechanisms, molecular mechanisms and adaptive strategies of *C. multiflorus* in response to drought is important for its growth in arid and semi-arid regions.

**Methods:**

We investigated the response and coupling mechanisms of water status, photosynthetic properties and chloroplast fluorescence parameters in *C. multiflorus* in response to drought and rehydrated after drought, especially the importance of nocturnal sap flow and nocturnal water refilling to maintain its own water balance in response to drought stress. In addition, we studied the stress response of *C. multiflorus* transcriptome factors, and we also discussed drought adaptation strategies of *C. multiflorus*.

**Results:**

*C. multiflorus* adapted to drought stress by a series of structural and physiological mechanisms, such as promoting closing stomata, increasing nocturnal sap flow. When rehydrated after undergoing severe drought stress, its physiological activities such as photosynthesis, water status, chlorophyll fluorescence parameters and other physiological activities have rapidly resumed. This showed *C. multiflorus* had strong tolerance to drought. In addition, water status, photosynthetic characteristics, and chloroplast fluorescence parameters of *C. multiflorus* were highly coupled. Nocturnal sap flow and nocturnal water refilling were very important for *C. multiflorus* to maintain its own water balance in response to drought stress. Finally, *C. multiflorus* will strengthen the drought defense mechanism by gene regulation of various metabolisms, such as promoting stomatal closure, reducing transpiration water loss, and vigorously regulating water balance. *C. multiflorus* responded to drought stress by avoiding or reducing water deficit in plant organs and tissues. Therefore, the shrub *C. multiflorus* is a drought-tolerant plant.

**Discussion:**

We explored the response mechanisms of water status, photosynthetic characteristics, and chloroplast fluorescence parameters of *C. multiflorus* in drought and rehydrated after drought stress, especially the response mechanisms of nocturnal sap flow and nocturnal water refilling in response to drought stress, and identified the physiological coupling mechanisms, molecular mechanisms and drought types of *C. multiflorus* in response to drought.

## 1 Introduction

As global temperatures rise and precipitation patterns change, droughts have become increasingly frequent in recent years (Réjou-Méchain et al., 2021; Naumann et al., 2018). The trend of extreme drought frequency is even more pronounced (Gu et al., 2023; Christian et al., 2023; Batibeniz et al., 2023). These frequent droughts have had significant impacts on the local carbon balance and forest ecosystem services (Hartmann et al., 2022; Anderegg et al., 2020), including causing large-scale tree mortality at a regional level (Choat et al., 2018; Goulden and Bales, 2019; Zhang et al., 2023). Therefore, the response and adaptation of plants to drought stress have become a focal point in ecological and plant physiological research (Werner et al., 2021; Anderegg et al., 2018; Yao et al., 2023; Vinod et al., 2023; Wang et al., 2023a).

The causes, climatic thresholds and modelling of drought-induced tree mortality have been well studied (Pereira et al., 2024; Wion et al., 2022; Valerian et al., 2021), however, the underlying physiological mechanisms are still a subject of debate (Martinez-Vilalta et al., 2019; Grossiord et al., 2020; Trugman et al., 2018). During short-term and severe droughts, water availability is often the primary factor influencing plant mortality, while during extended periods of drought, the significance of carbon availability for critical survival functions tends to become more prominent (Arend et al., 2021; Wang et al., 2020). Nevertheless, it is essential to minimize the blockage of xylem conduits in order to enhance the transport of water to the canopy and regenerative tissues (apical and formative meristematic tissues) of plants. Among these mechanisms, stomatal regulation is the most crucial in preventing xylem embolism by regulating water transport and leaf transpiration (Chen et al., 2022). It is worth noting that the nighttime fluid flow has the effect of nighttime transpiration and stem hydration, and also helps to reduce the formation of xylem embolism and gas holes in plants, and avoid hydraulic failure and even death of plants due to drought (Klein et al., 2018; Wu et al., 2023). This is because night transpiration transporting the water replenishment of stems improves the low water potential of stems caused by daytime transpiration, prevents xylem embolism, repairs air holes to a certain extent, and avoids the death of plants caused by frequent hydraulic failure (Klein et al., 2018; Zeppel et al., 2019). In arid zones, these mechanisms are especially crucial for plant survival.

With global changes leading to shifts in climate patterns, the vegetation in arid zones is typically dominated by small trees, shrubs, or semi-shrubs, which act as pioneer species adapted to harsh conditions. These plants rely on efficient water transport mechanisms to withstand the challenges of arid environments and maintain their survival in the face of changing environmental conditions (Shen et al., 2022; Zhou et al., 2022; Davis et al., 2020). *Cotoneaster multiflorus* is a shrub belonging to the *Cotoneaster* genus in the apple subfamily (Maloideae) of the rose family (Rosaceae). This species is known for its robust characteristics, including a large root system, and its ability to withstand cold temperatures, drought conditions, and poor soil fertility. These traits make it well-suited for challenging environments and highlight its adaptability and resilience in adverse growing conditions (Chen et al., 2021). *C. multiflorus* is a pioneering species for vegetation restoration in the ecological transition zones of arid and semi-arid loess hills and gullies and bedrock hills (tufaceous mountainous areas), where it is extremely widespread. The distribution of *C. multiflorus* populations continue to expand under various scenarios in different future climates (Huang et al., 2024). Meanwhile, *C. multiflorus* has high ecological benefits, such as wind prevention and sand fixation, water, soil conservation (Zhao et al., 2022) and ornamental role (Yang et al., 2022). It also has great economic benefits, such as medicinal development (Chang and Jeon, 2003; Liu et al., 2018; Jia, 2022) and as a grafting rootstock for *Malus pumila* Mill (Wang et al., 1983). Therefore, to study leaf water status, photosynthetic characteristics and stem fluid flow of *C. multiflorus* can help to reveal the response mechanism of the shrub to the environment in arid and semi-arid zones.

However, plant adaptation to drought stress is not attributed to a single trait but is instead a combination of various traits that work together and are correlated with each other (McDowell et al., 2022; López et al., 2021). For example, plants may adjust photosynthetic physiological traits (reducing stomatal conductance, increasing water use efficiency, and reducing transpiration rate) and hydraulic traits (reducing xylem hydraulic conductivity and conduit diameter, etc.) to reduce water loss in response to drought conditions (Sevanto et al., 2014; Vastag et al., 2020). Therefore, further research is needed to explore the relationships among different traits in order to better understand the adaptive mechanisms and coping strategies that plants employ to tolerate drought stress.

At present, the coping strategies and molecular mechanism of *C. multiflorus* under drought stress are not well known. The research involved analysis of 25 different indicators and high-throughput sequencing to assess patterns of water status, photosynthetic characteristics, trunk runoff, and transcription factor in response to different drought intensities and rehydrated processes. Through the use of integrated analyses such as correlation analysis, principal component analysis, DEGs analysis and GO analysis, the study sought to provide insights into the adaptive strategies employed by *C. multiflorus* to withstand drought stress. The findings were expected to enhance our understanding of the growth patterns of *C. multiflorus* in arid and semi-arid regions and elucidate the strategies utilized by the species to cope with drought conditions, and further exploration of its drought resistance mechanism. The results of this study will improve our understanding of coping strategies under drought stress conditions and enhance the management of this important woody species in arid regions.

## 2 Materials and methods

### 2.1 Test materials and moisture control

The State Key Laboratory of Seedling Bioengineering (Ningxia Forestry Research Institute Co., Ltd.) provided 2-year-old *C. multiflorus* with robust growth, free of pests and diseases, and basically the same growth. On May 1, 2023, 350 pots (1 plant per pot) of *C. multiflorus* seedlings were cultivated in the greenhouse of Fujian Agriculture and Forestry University. The plastic pots used for the experiment were 20.5 cm (calibre) × 14.5 cm (ground diameter) × 18.5 cm (height). The potting soil was yellow soil, soil weight was 4.0 Kg/pot, in which the soil organic carbon (SOC), organic matter (SOM), available nitrogen (AN), available phosphorus (AP) and available potassium (AK) were 7.22 (g/Kg), 12.44 (g/Kg), 70.00 (mg/Kg), 7.90 (mg/Kg) and 7.72 (mg/Kg), respectively. During the experiment, the average daytime temperature and nighttime temperature were 38.8°C and 30.6°C, respectively, and the average daytime humidity and nighttime humidity were 52.5% and 69.1%, respectively.

From July 25 to 31, 2023, 350 test seedlings were subjected to different degrees of water deficit cultivation and partial normal water management. On August 1, 2023, 150 healthy *C. multiflorus* seedlings without diseases and insect pests, with similar growth potential and different water deficits were selected as experimental materials. The experiment was set up with 4 drought level treatments and 1 rehydrated treatment, a total of 5 treatments, each treatment was repeated 3 times, 10 plants per replicate, that was, 30 plants per treatment. Specific operations are as follows:

According to the soil relative water content (WCH) of 60% ~ 80%, 40% ~ 60%, and 20% ~ 40%, 30 seedlings were selected from 150 test seedlings as comparison (CK)、mild drought stress (LD)、moderate drought stress (MD), respectively. Finally, 60 plants with a WCH of 5% ~ 10% were selected. Among them, 30 plants were used as severe drought stress (SD), and the other 30 plants were replenished with water on August 6, 2023 and used as rehydrated treatment (RD).The assessment of each index began on August 8, 2023 and ended on August 10. Therefore, the drought stress time of CK, LD, MD, and SD was 7∼10 days, and the rehydrated lasts for 2∼5 days.

During the treatments, lost water was replaced daily by potting water control method (Luo et al., 2021; Zou et al., 2022), the pots were weighed and watered every 24h to maintain the corresponding soil moisture. The calculation formula of WCH is as follows (Wu, 2020):


(1) $$WCH(\%)=(W_{1}-W_{2})/W_{2}\times 100$$

WCH % is the relative water content of soil, $W_{1}$ is the original soil quality, and $W_{2}$ is the soil quality after drying.

#### 2.2.1 Determination of leaf water potential and relative water content

The WP4C Dew Point PotentiaMeter was employed to measure the leaf water potential of *C. multiflorus* between 5:00 and 6:30 local time. Within each treatment group, 9 C*. multiflorus* plants were randomly chosen for analysis. From each selected plant, 10 mature leaves located in the middle of the plant were picked. In these 10 leaves, 5 were utilized to determine the leaf water potential using the WP4C Dew Point PotentiaMeter (Miao et al., 2017), the other 5 mature leaves were used to determine the relative water content of the leaves by the saturated weighing method (RWC) (Wang et al., 2023b).

#### 2.2.2 Leaf stomatal determination

In the experiment, 3 plants of *C. multiflorus* from each treatment were randomly chosen. From each selected plant, 3 mature leaves were then randomly selected for stomatal determination. Stomatal characteristics were observed in 10 randomly selected fields of view of each leaf using a bench electron microscope (TM3030 Plus). The measurements of stomatal features including stomatal opening length, stomatal opening width, stomatal aperture, and stomatal density were obtained using ImageJ software. This analysis aimed to provide insights into the stomatal behavior of *C. multiflorus* under varying drought stress levels.

#### 2.2.3 Trunk sap flow measurement

From August 9 to August 31, 2023, based on preliminary survey of sapwood thickness of *C. multiflorus* (Leng, 2020), each of the 12 C*. multiflorus* plants with similar growth was fitted with a probe (probe length 10 mm, probe diameter 1.2 mm). The probe was connected to the i-MIX data collector and the average value was calculated every 5 min and automatically stored in the data collector. Throughout the monitoring period, the soil water content was consistently monitored daily. The *C. multiflorus* experienced 3 times from the initial watering, to severe drought stress.

Additionally, the temperature variance between thermocouples was tracked using a probe to calculate the heat dissipation associated with sap flow. By establishing a relationship between the temperature difference and sap flow rate, the size of the sap flow rate was determined. This methodology aimed to provide insights into the plant’s response to varying soil moisture levels and their ability to adjust to changing environmental conditions.

Based on the observed data, Granier’s empirical formula (Granier, 1987) was used to derive the instantaneous trunk sap density $F_{\text{d}}$ (g/(m²·s)) :


(2) $$F_{\text{d}}=119\times {\left(\frac{\Delta T_{\max}-\Delta T}{\Delta T}\right)}^{1.231}$$

Among them, ΔT (°C) is the instantaneous temperature difference between day and night; $\Delta T_{\max}$ (°C) is the maximum instantaneous temperature difference between day and night. In general, at the time of maximum temperature difference, the density of nighttime trunk sap flow was zero.

#### 2.2.4 Measurement of photosynthesis parameters

In each treatment group of 30 plants of *C. multiflorus*, three mature leaves were selected from each plant. The Li-6800 portable photosynthesis meter (LI-COR, USA) was used to measure the photosynthetic rate of plant leaves ( $P_{n}$ ), stomatal conductance ( ${\text{G}}_{s}$ ), transpiration rate( $T_{r}$ )at the time of the day (9:00-11:00). And 3 replicates were performed for each leaf. Among them, 6800-01A chamber was used to determine the photosynthetic index; the carbon dioxide concentration in the reference chamber was set to 400 μmol·mol^-1^, RHcham was 67.94%, and flow rate was 500 μmol·s^-1^. Water use efficiency (WUE) calculation formula is as follow (Yan et al., 2022) :


(3) $$WUE=P_{n}/T_{r}$$

#### 2.2.5 Rapid determination of chlorophyll fluorescence parameters

In each treatment group of 30 C*. multiflorus* plants, 3 mature leaves were selected from each plant, and then chlorophyll fluorescence parameters were determined using a FluorPen (FP100) hand-held fluorometer, and the leaves were dark-adapted for 15 min before the determination (Ghaffar et al., 2023).

### 2.3 Transcriptome determination and analysis

#### 2.3.1 Total RNA extraction and cDNA library construction

The young leaves of *C. multiflorus* of CK, SD and RD test groups were removed, frozen and ground with liquid nitrogen quickly. The total RNA of the samples was extracted by Trizol method. The integrity and purity of RNA was analyzed by agarose gel electrophoresis and ultra-micro spectrophotometer. Finally, the concentration and quality of RNA were measured by fluorescence quantifier.

2 μg RNA samples were taken to construct cDNA Library, using NEBNext^®^ UltraTM RNA Library Prep Kit for Illumina kit (New England Biolabs). AMPure XP beads (Be control group man Coulter) were used to purify the cDNA samples (size between 100 ~ 200 bp), and the quality of the library was evaluated by Agilent 2100 biological analyzer.

#### 2.3.2 Transcriptome sequencing and data quality control

After the quality inspection of the cDNA library was qualified, the different libraries were mixed according to the requirements and RNA-Seq sequencing was performed using the DNBSEQ-T7 sequencing platform. The obtained RNA sequencing data were subjected to quality control filtering through the fastp v0.19.3 platform (Vinod et al., 2023). The specific filtering criteria are as follows: 1) reads with adapters are not retained; 2) a read with N content more than 10% of its own base number is not retained; 3) a read with the number of low-quality bases more than 50% of its own base number is not retained. After quality control, clean reads were obtained for subsequent transcriptome analysis.

#### 2.3.3 Clean reads comparison of reference genomes

Based on the HISAT2 software (v2.2.1) (Valeriano et al., 2021), the obtained clean reads were aligned to the assembled *C. multiflorus* genome. The software parameters were set by default.

#### 2.3.4 Analysis of gene expression levels and differences

The FPKM (Fragments Per Kilobase of exon model per Million mapped fragments) for each gene was calculated using feature Counts v1.6.2 software (Wion et al., 2022). A single gene with FPKM ≥ 1 was retained as the screening basis for subsequent gene differential expression. The DESeq2 v1.22.1 package of R platform was called to perform gene differential expression analysis (Jung et al., 2023).

#### 2.3.5 Functional annotation and GO enrichment analysis of differentially expressed genes

GO enrichment analysis was mainly composed of molecular function, biological process and cellular component. The obtained non-redundant transcript sequence was subjected to GO enrichment analysis using cluster Profiler (v4.0.5) (Martinez et al., 2019). Benjamini-Hochberg method was used as a multiple test method, and GO term with FDR < 0.05 was defined as significant enrichment.

### 2.4 Data processing

Data were organized using Microsoft excel 2019 software. Correlation analysis and principal component analysis were performed using DPS 7.5. ANOVA with multiple comparisons (Ducan’s method) were performed using SPSS. Graphs were plotted using Origin. Data used in the graphs were mean ± standard error.

## 3 Results

### 3.1 Effect of drought stress and rehydrated on water potential and relative water content (RWC) of *C. multiflorus* leaves

**Figure 1:**
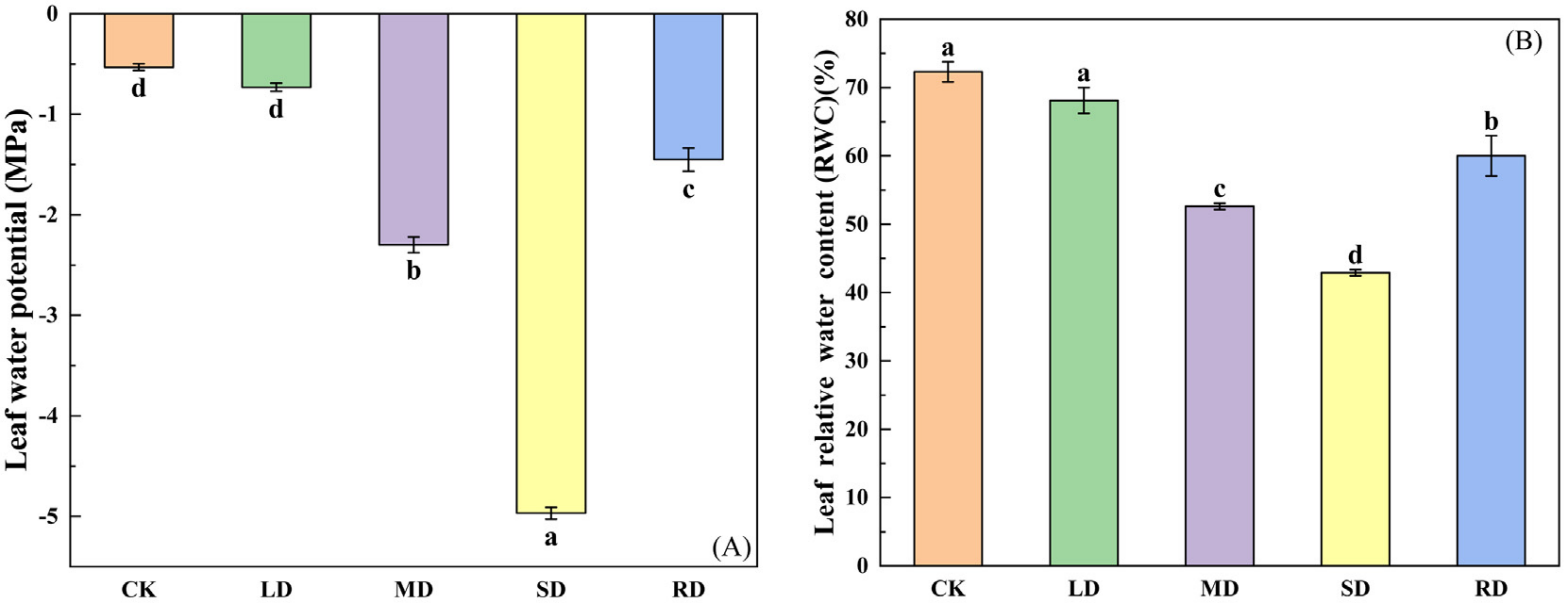
Effects of drought stress and rehydrated on leaf water potential and RWC of *C. multiflorus.* There was a significant difference between the different English letters marked in the figure (P < 0.05).

Both of them showed a downward and then an upward trend, leaf water potential decreased by -0.21 MPa, -1.77 MPa and -4.44 MPa under mild drought (LD), moderate drought (MD) and severe drought stresses (SD), respectively, compared to the control group (CK). After RD treatments, the leaf water potential of *C. multiflorus* increased by 0.85 MPa and 3.52 MPa compared to MD and SD, respectively (**Figure 1A**). Relative water content (RWC) decreased by 5.79%, 27.23% and 40.68%, respectively, compared to the CK (**Figure 1B**). After RD treatments, leaf RWC of *C. multiflorus* was elevated by 39.91% and 14.05%, respectively, compared to MD and SD.

### 3.2 Effect of drought stress and rehydrated on stomatal characteristic of *C. multiflorus* leaves

**Figure 2:**
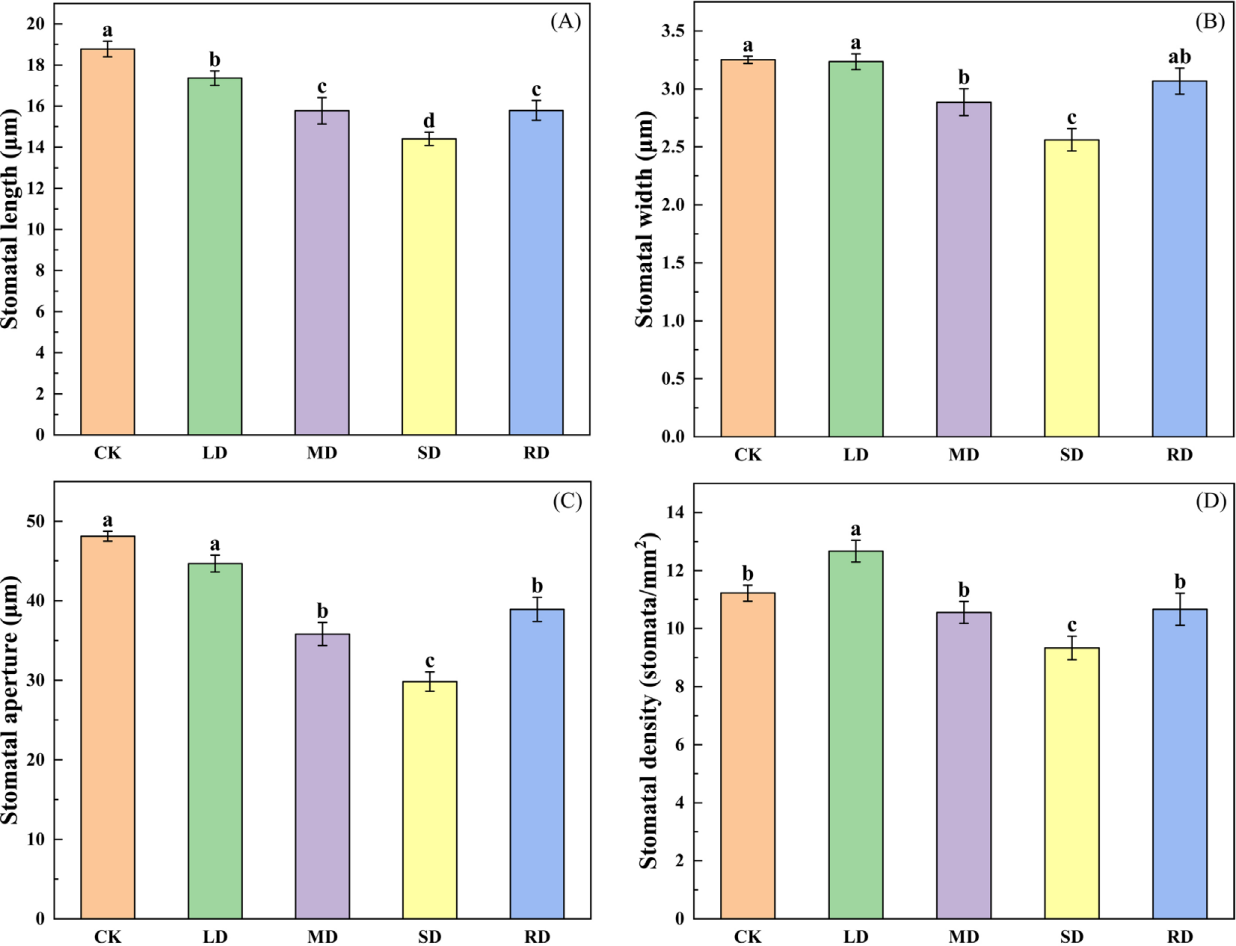
Effects of drought stress and rehydrated on stomatal characteristic of *C. multiflorus.* There was a significant difference between the different English letters marked in the figure (P < 0.05).

As can be seen in **Figure 2**, as the degree of drought increased, the stomatal opening length, stomatal opening width, stomatal aperture and stomatal density (except LD) of *C. multiflorus* leaves were significantly reduced. Under SD, the above-mentioned variables were reduced by 23.27%, 21.22%, 37.86%, 16.83% and 48.71%, respectively, compared to the control group (CK). However, After RD treatments (48 h), those variables were able to return to the state between LD and MD.

### 3.3 Effect of drought stress and rehydrated on stem sap flow of *C. multiflorus*

#### 3.3.1 Diurnal variation of stem sap flow of *C. multiflorus* under drought stress and rehydrated

**Figure 3:**
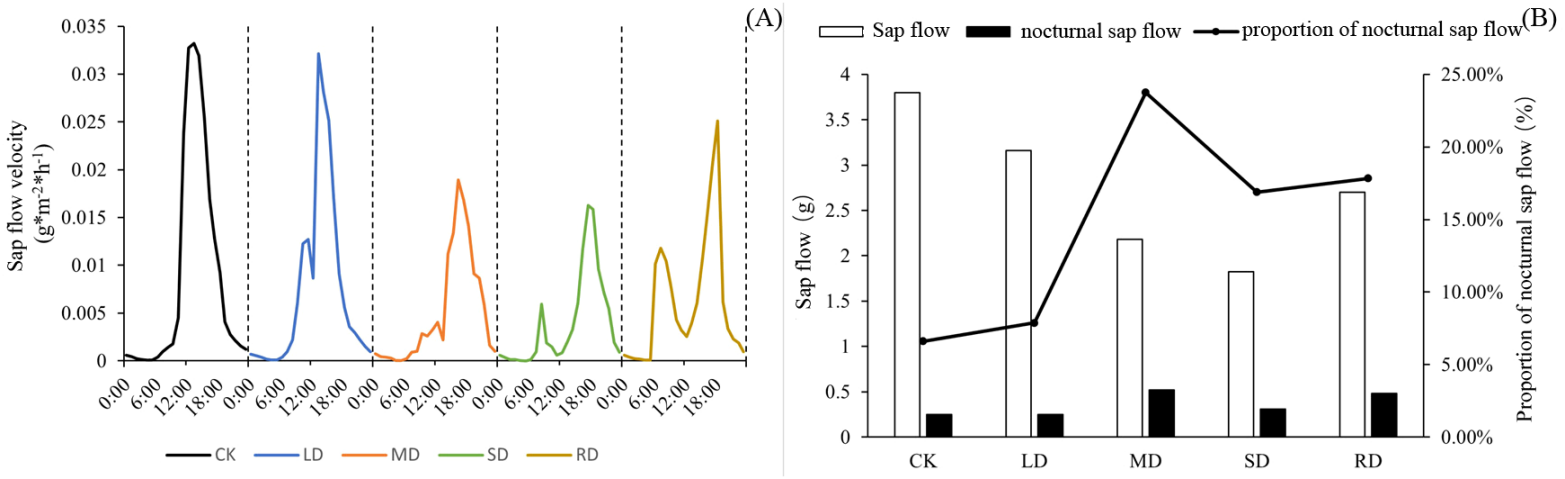
Diurnal variation of sap flow of *C. multiflorus* under drought stress and rehydrated.

**Figure 3A** showed that *C. multiflorus* had a ‘lunch break’ from LD. The diurnal variation of stem sap flow rate appeared double or multiple peaks. As the degree of drought stress increased, the first peak appeared earlier and earlier. Under LD, MD and SD, the first peak of *C. multiflorus* appeared at about 11:00, 10:00 and 8:00, respectively. Even after RD treatments, *C. multiflorus* was not able to return to its initial state in a short time, and the phenomenon of ‘double peak’ still existed.

C. *multiflorus* showed a gradual decrease in all-day sap flow from LD. The opposite was true for nocturnal sap flow, which gradually increased with increasing drought stress levels, in the case of SD, nocturnal sap flow, on the other hand, was significantly lower than in the case of MD. However, after RD treatments, both all-day and nocturnal sap flow were elevated (**Figure 3B**). There was little difference in the ratio of nocturnal sap flow to daily between the LD and CK, which were 6.59% and 7.87%, respectively. However, this index at MD and SD was 2.60-fold and 1.56-fold higher than that of the CK. After RD treatments, the nocturnal sap flow of *C. multiflorus* accounted for 5.69% higher than that under SD.

#### 3.3.2 Distributional characteristics of nocturnal sap flow of *C. multiflorus* under drought stress and rehydrated

**Figure 4:**
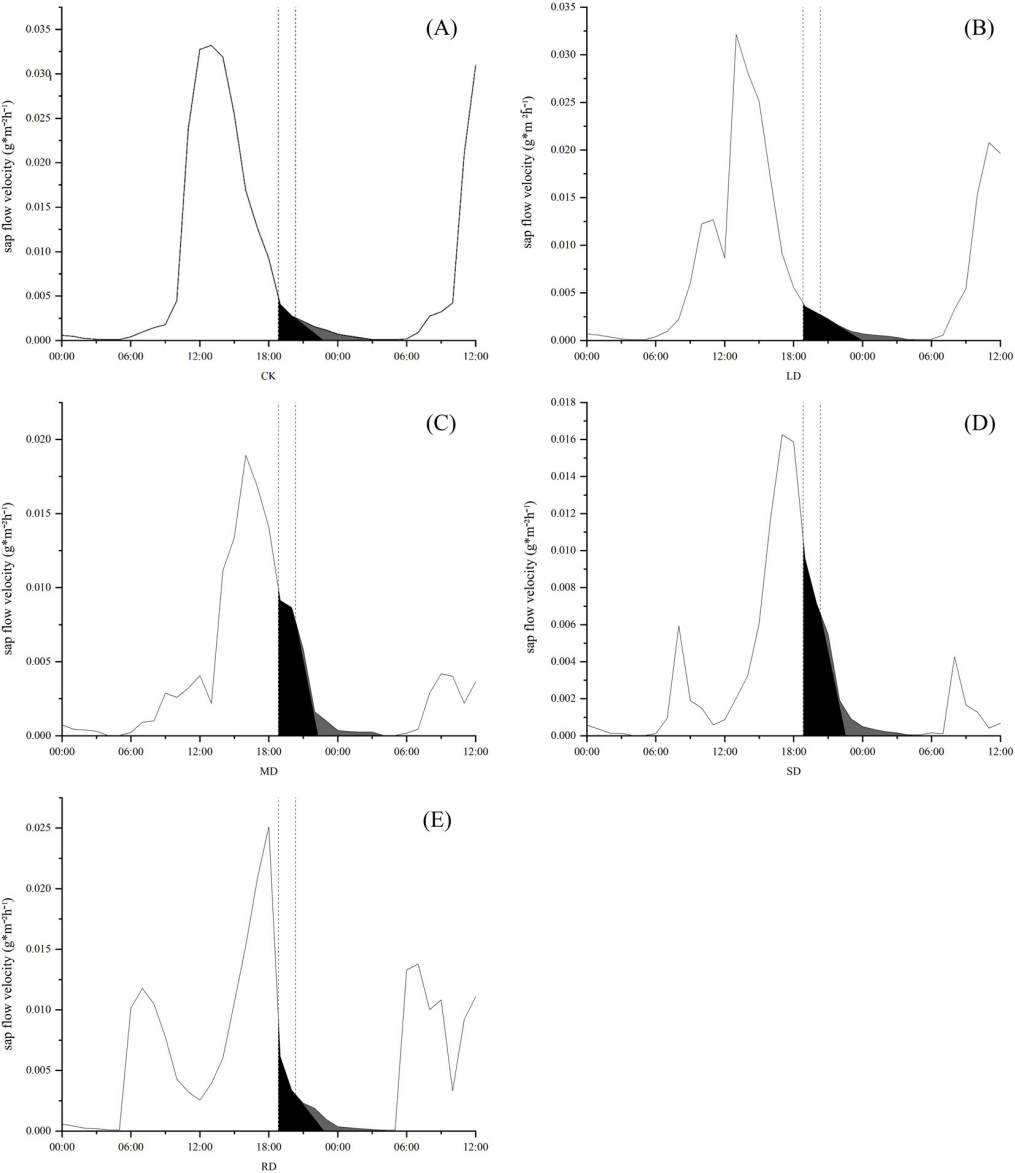
Distributional characteristics of sap flow of *C. multiflorus* under drought stress and rehydrated. The spacing between the two vertical gray dashed lines represents the lag time (80 minutes,18:50∼20:10) of sap flow activity and solar radiation changes. The black shaded area is nighttime stem rehydrated, and the gray shaded area is nighttime transpiration.

Based on the integral area of each region (**Figure 4**), it was clear that *C. multiflorus* has both nocturnal water refilling and nocturnal transpiration at night. *C. multiflorus* responded rapidly to nocturnal water refilling under different drought stresses, increasing by 14.25%, 25.53% and 29.34%, respectively, compared to CK. The nocturnal water refilling under SD increased by only 3.04% compared to that under MD. But after RD treatments, the nocturnal water refilling of *C. multiflorus* was 7.50% lower than that under SD. Meanwhile, nocturnal transpiration of *C. multiflorus* gradually decreased with increasing drought stresses, accounting for 30.22%, 20.27%, 10.37%, 9.74% and 16.50% of nocturnal sap flow, respectively.

### 3.4 Effect of drought stress and rehydrated on photosynthetic characteristics of *C.multiflorus*

**Figure 5:**
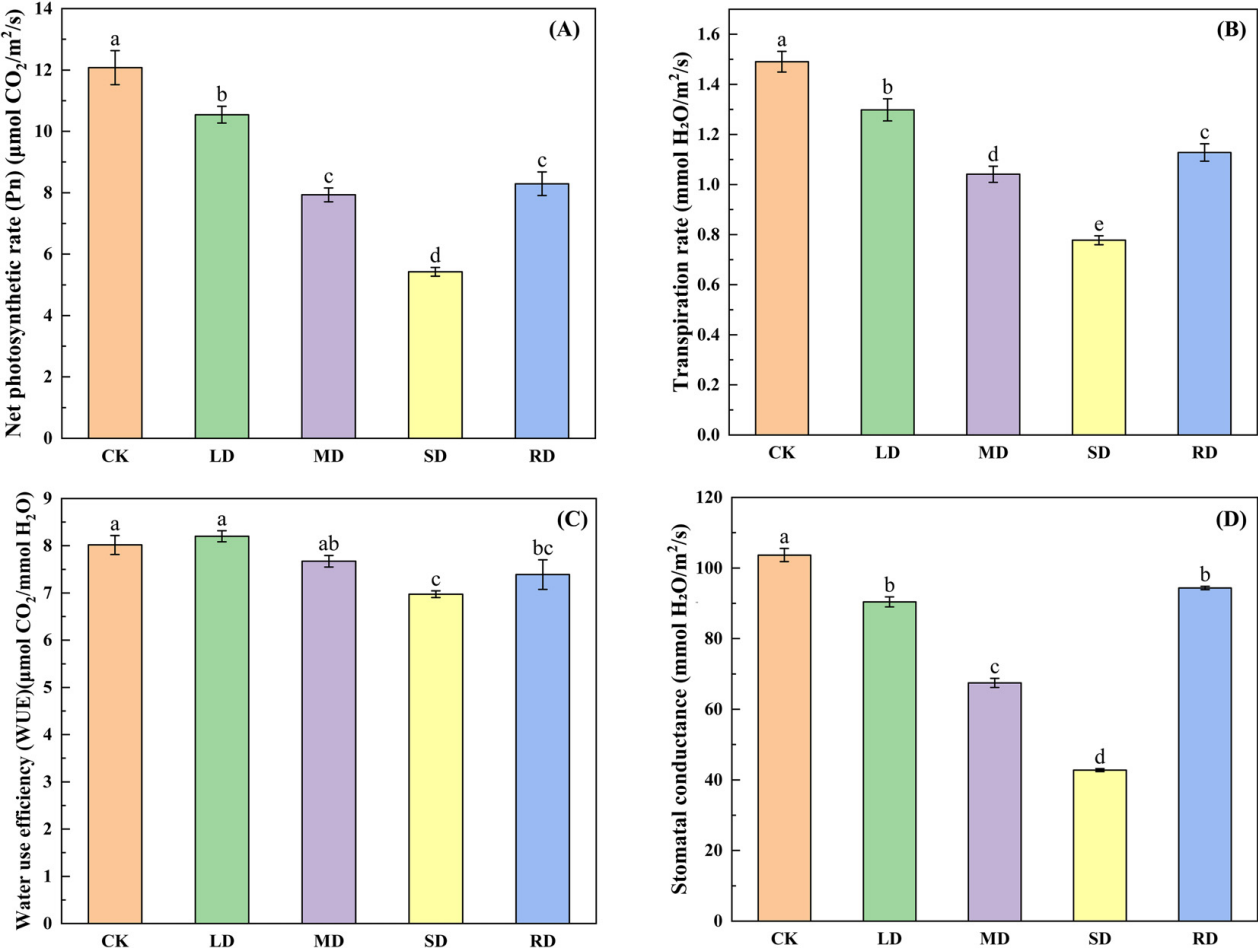
Effects of drought stress and rehydrated on photosynthetic characteristic of *C. multiflorus*. There was a significant difference between the different English letters marked in the figure (P < 0.05).

As can be seen from **Figure 5**, the $P_{n}$ , $G_{\text{s}}$ , and $T_{r}$ tended to decrease with increasing stress levels in all drought stress treatments, with $P_{n}$ decreasing by 12.73%, 34.32%, and 55.08% under LD, MD, and SD, respectively, compared with the CK. The $G_{\text{s}}$ decreased by 12.67%, 34.81% and 58.71%, while $T_{r}$ decreased by 12.52%, 29.84% and 47.60%, respectively, compared to the CK. The water use efficiency (WUE) under LD increased by 0.26% against the CK, while WUE under MD and SD decreased by 5.39% and 13.99% against the CK, respectively. After RD treatments, the $P_{n}$ , $G_{\text{s}}$ , $T_{r}$ , and WUE showed an increasing trend, which increased by 52.87%, 118.84%, 45.12%, and 6.43% compared to the CK.

### 3.5 Effect of drought stress and rehydrated on chlorophyll fluorescence parameters of *C. multiflorus*

#### 3.5.1 Changes in PSII reaction centers

**Figure 6:**
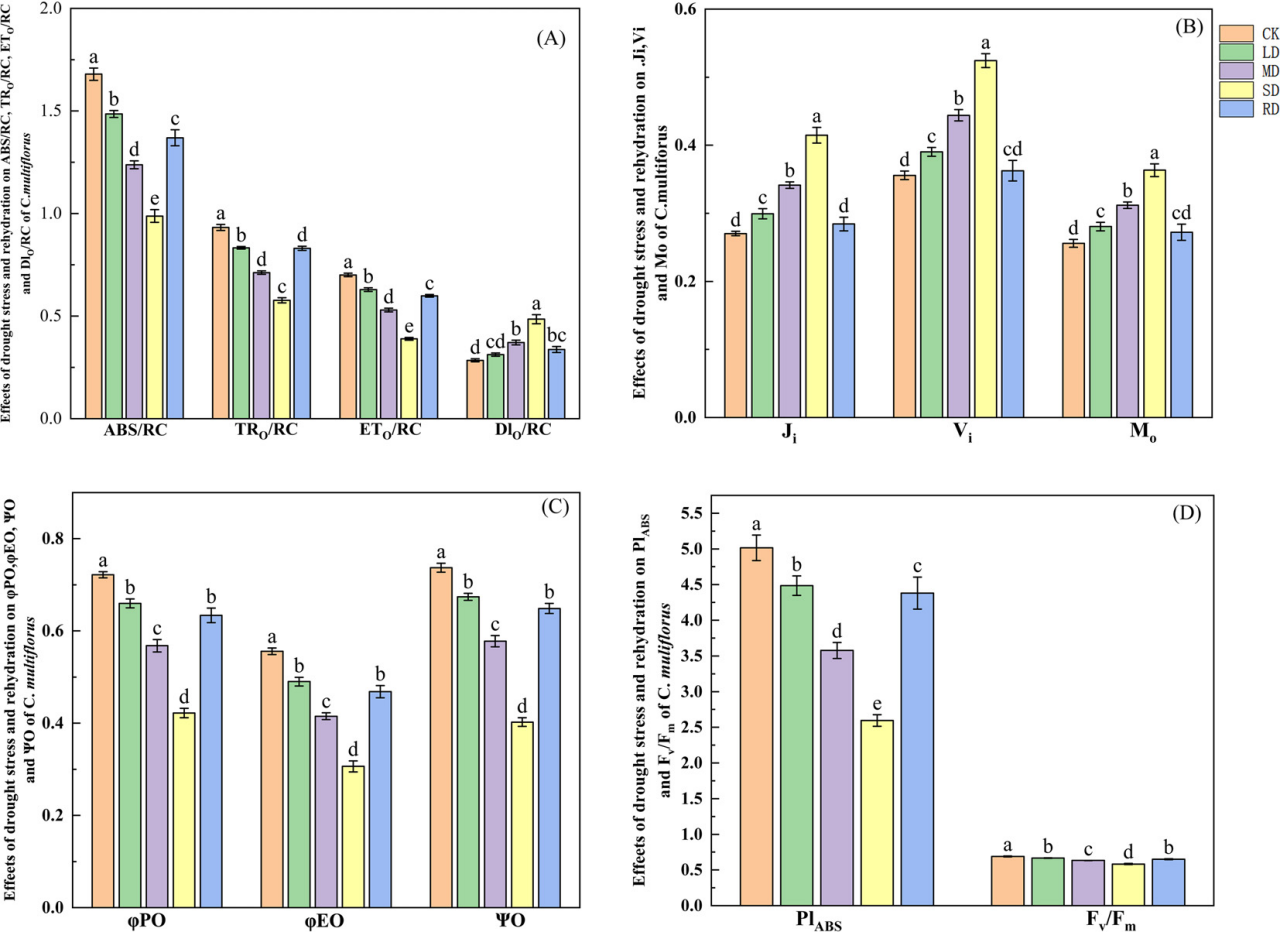
Effects of drought stress and rehydrated on chlorophyll fluorescence of *C. multiflorus*. There was a significant difference between the different English letters marked in the figure (P < 0.05).

From **Figure 6A**, the ABS/RC decreased by 11.55%, 26.29% and 41.16% under LD, MD and SD, respectively, compared to the CK. The $TR_{O}/RC$ decreased by 10.62%, 23.65% and 38.12%, respectively, compared to the CK. The $ET_{O}/RC$ decreased by 10.23%, 24.48% and 44.45%, respectively, compared to the CK. After RD treatments, the ABS/RC , $TR_{O}/RC$ and $ET_{O}/RC$ were elevated by 38.58%, 44.43% and 52.93%, respectively, compared to SD. The energy dissipated by heat ( $DI_{O}/RC$ ) showed a tendency to increase and then decrease, with $DI_{O}/RC$ elevated by 9.92%, 30.51% and 70.58%, respectively, compared to the CK. It reduced by 30.51% after rehydrated, compared to SD.

#### 3.5.2 Changes on the Acceptor Side of PSII

From **Figure 6B**, the $M_{O}$ increased by 9.57%, 21.80% and 41.95% under LD, MD and SD, compared to CK. The $J_{i}$ increased by 9.67%, 24.79% and 47.36%, respectively, compared to CK. The $V_{\text{i}}$ increased by 10.69%, 26.27% and 53.42%, respectively, compared to CK. After RD treatments, $M_{O}$ , $J_{i}$ and $V_{\text{i}}$ decreased by 25.10%, 30.84% and 31.44%, respectively, compared to that of SD. From **Figure 6C**, the $\phi_{PO}$ decreased by 8.62%, 21.33% and 41.54% under LD, MD and SD, respectively, compared to CK. The $\phi_{EO}$ decreased by 11.85%, 25.33% and 44.83% respectively, compared to CK. The $\psi_{O}$ decreased by 8.55%, 21.62% and 45.43% respectively, compared to CK. After RD treatments, $\phi_{PO}$ , $\phi_{EO}$ , $\psi_{O}$ and $\psi_{O}$ increased by 50.19%, 52.76% and 61.25% respectively, compared to that of SD.

#### 3.5.3 Changes in blade performance index and $F_{v}/F_{\text{m}}$

From **Figure 6D**, the $PI_{ABS}$ decreased by 10.58%, 28.70% and 48.25% under LD, MD and SD, respectively, compared to CK. The $F_{v}/F_{\text{m}}$ decreased by 3.39%, 8.31% and 15.60%, respectively, compared to CK. After RD treatments, $PI_{ABS}$ and $F_{v}/F_{\text{m}}$ increased by 68.80% and 11.65% respectively, compared to that of SD. It can be seen that the photosynthetic performance index $PI_{ABS}$ of *C. multiflorus* leaves was more sensitive to drought stress than $F_{v}/F_{\text{m}}$ .

### 3.6 Correlation analysis of water status and photosynthetic characteristics of *C. multiflorus* under drought stress and rehydrated

**Figure 7:**
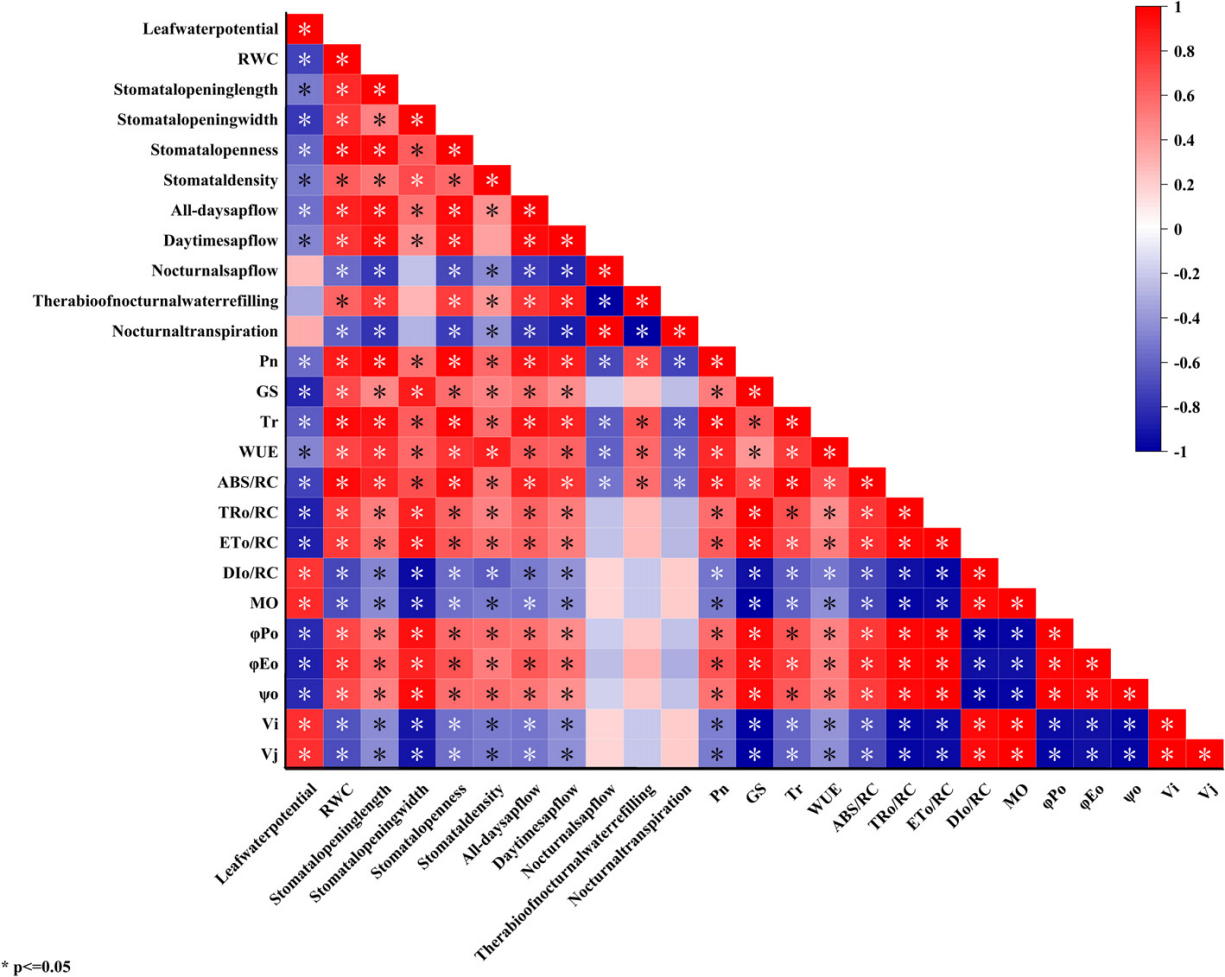
Correlation analysis of water status and photosynthetic characteristics of *C. multiflorus* under drought stress and rehydrated.

As can be seen from **Figure 7**, there was a significant or extremely significant positive correlation between the water status indicators of *C. multiflorus* such as leaf water potential, RWC, average stomatal length, average stomatal width, average stomatal aperture, all-day sap flow, daytime sap flow and the photosynthetic characteristics indicators including net $P_{n}$ , stomatal conductance, $T_{\text{r}}$ , WUE, ABS/RC , $TR_{O}/RC$ , $ET_{O}/RC$ , $\phi_{PO}$ , $\phi_{EO}$ , and $\psi_{O}$ . There was a significant or highly significant negative correlation with both $DI_{O}/RC$ and $M_{O}$ . Leaf water potential, RWC, mean stomatal width, mean stomatal aperture and all-day sap flow were significantly or highly significantly negatively correlated with $V_{i}$ and $J_{i}$ . Mean stomatal length was negatively and significantly negatively correlated with $V_{i}$ and $J_{i}$ , respectively. Mean stomatal density and daytime sap flow were negatively correlated with $V_{i}$ and $J_{i}$ .

Significantly, a positive correlation was observed between the ratio of nocturnal water refilling and various plant parameters including leaf water potential, leaf relative water content, mean stomatal length, mean stomatal width, mean stomatal aperture, and mean stomatal density. Additionally, there was a notable positive correlation between the ratio of nocturnal water refilling and all-day as well as daytime sap flow. Interestingly, a significant negative correlation was found between the ratio of nocturnal water refilling and nocturnal sap flow. The ratio of nocturnal water refilling was positively correlated with the photosynthetic characteristics of $P_{n}$ , stomatal conductance, $T_{r}$ , WUE, ABS/RC , $TR_{O}/RC$ , $ET_{O}/RC$ , $\phi_{PO}$ , $\phi_{EO}$ , $\psi_{O}$ and negatively correlated with $DI_{O}/RC$ , $M_{O}$ , $V_{i}$ and $J_{i}$ .

### 3.7 Principal component analysis of indicators of water status and photosynthetic characteristics of *C. multiflorus* under drought stress and rehydrated

The 25 drought tolerance indicators of *C. multiflorus* under different drought levels and rehydrated states were analyzed by principal component analysis. The first 2 principal components had a cumulative contribution of 96.351% and an eigenvalue > 1, which was mainly representative (**Table 1**). The contribution rate of principal component I was 86.366%, and the loadings of the other 19 indicators in principal component I were 0.2022∼0.2148 (including negatively correlated indicators). The contribution of principal component II was 9.9856%, the loadings of nocturnal sap flow, the ratio of nocturnal water refilling and nocturnal transpiration on principal component II were higher, indicating that principal component II mainly reflected nocturnal water refilling and nocturnal transpiration of *C. multiflorus* under drought stress (**Supplementary Table 1**).

**Table 1:** Eigenvalue of two principal components and contributions.

Principal	Eigen value	Contribution (%)	Cumulative contribution (%)
1	21.5914	86.3658	86.3658
2	2.4964	9.9856	96.3513

### 3.8 DEGs analysis and GO analysis under drought stress and rehydrated of *C. multiflorus*

**Figure 8:**
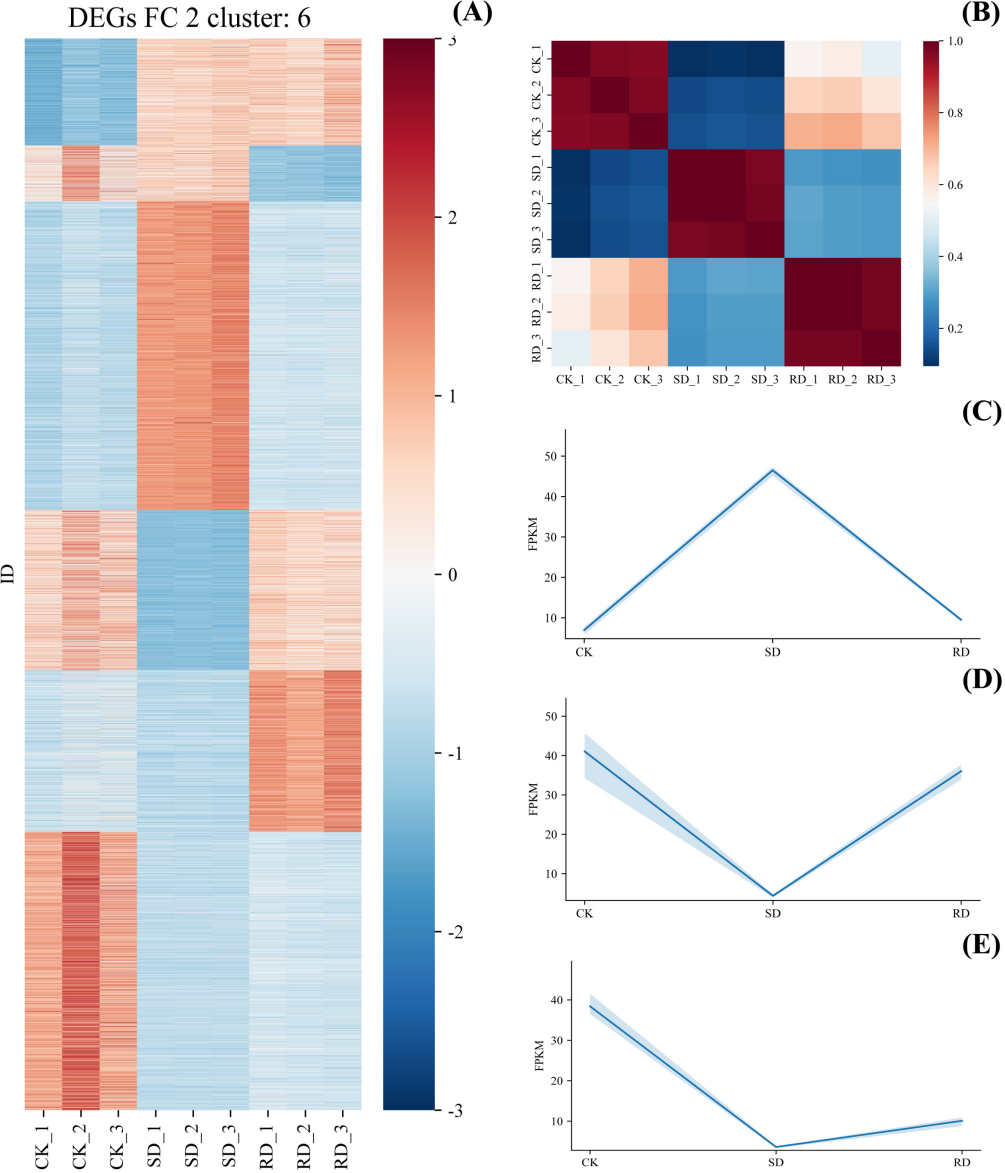
Spearman correlation analysis and DEGs cluster analysis of RNA expression in (C) *multiflorus* leaves under drought stress and rehydrated. **(A)** DEGs cluster analysis; **(B)** Spearman correlation analysis; **(C)** linear fitting of the second category; **(D)** linear fitting of the third category; **(E)** linear fitting of the fifth category.

The Spearman correlation of the leaves of *C. multiflorus* was calculated by selecting the genes with mean FPKM expression greater than 1. As shown in **Figure 8B**, the correlation between different treatments was high, indicating that the biological repetition was reliable. All DEGs (FC > 1, *P* < 0.01) were further processed by Z-Scrae method, and then cluster analysis was performed by machine learning K-Means method (parameter n = 5). A total of 5 categories were clustered (**Figure 8A**). Among them, in the second category (**Figure 8C**), the expression of DEGs was significantly up-regulated in SD, and was significantly down-regulated in RD. In the third category and the five categories (**Figure 8D, E**), the opposite was true, indicating that these three types of DEGs were related to drought in *C. multiflorus*.

**Figure 9:**
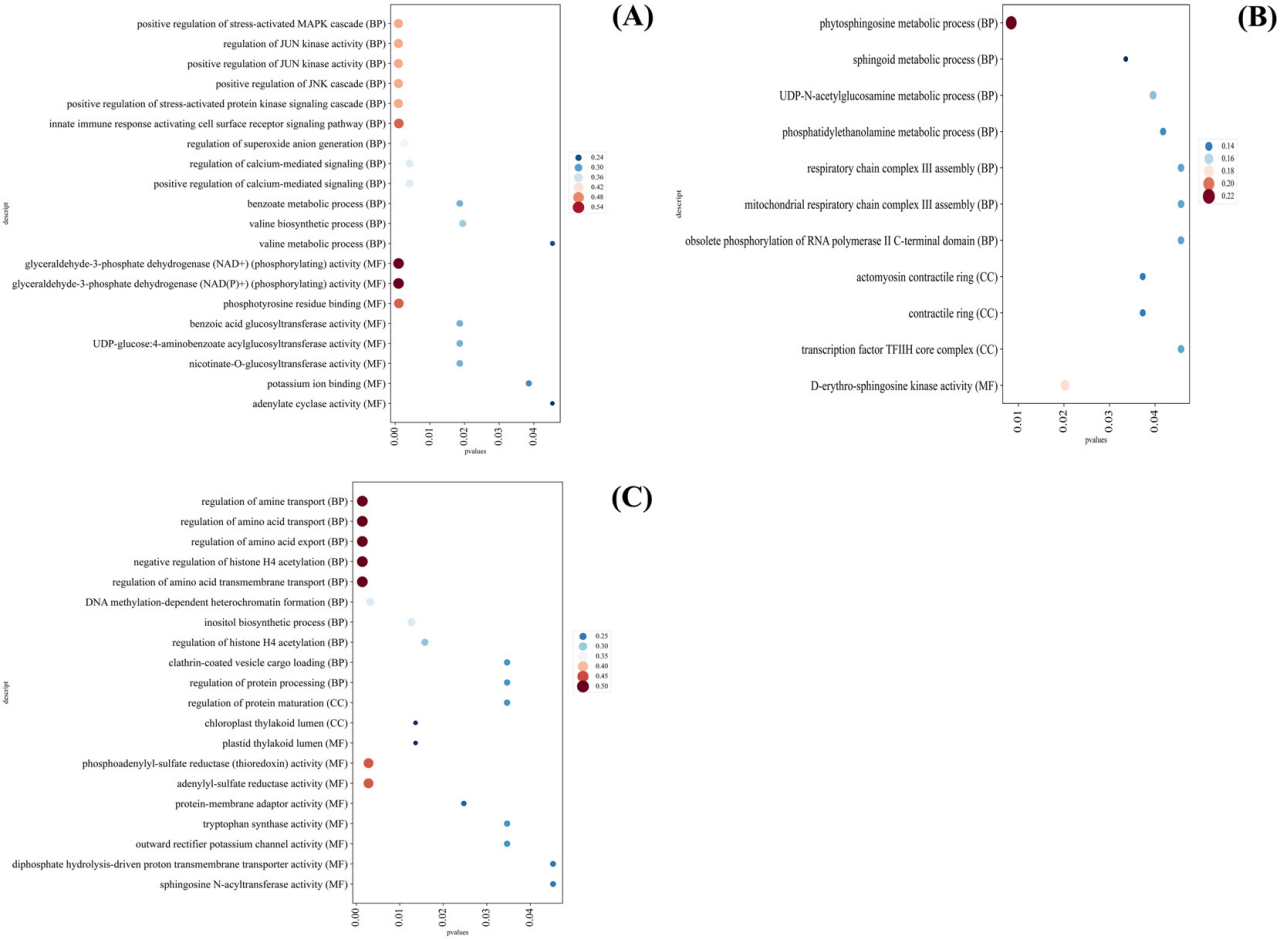
GO analysis of different categories of *C. multiflorus* leaves under drought stress and rehydrated. **(A)** GO analysis of the second category; **(B)** GO analysis of the third category; **(C)** GO analysis of the fifth category.

GO analysis showed that the GO terms with significantly up-regulated DEGs expression in SD were mainly enriched in positive regulation of stress-activated MAPK cascade, regulation of JUN kinase activity, positive regulation of JUN kinase activity, positive regulation of JNK cascade, positive regulation of stress-activated protein kinase signaling cascade and innate immune response activating cell surface receptor signaling pathway (**Figure 9A**).

GO terms with significantly down-regulated DEGs expression in SD were mainly enriched in phytosphingosine metabolic process and D-erythro-sphingosine kinase activity (**Figure 9B**), regulation of amine transport, regulation of amino acid transport, regulation of amino acid export, negative regulation of histone H4 acetylation, and regulation of amino acid transmembrane transport (**Figure 9C**).

## 4 Discussion

### 4.1 Effect of drought-rehydrated on water status of *C. multiflorus*

With the intensification of drought stress, leaf water potential (**Figure 1A**), RWC (**Figure 1B**) and Tr (**Figure 4**) of *C. multiflorus* decreased significantly under different drought stresses. This is the same as the findings of other scholars (Song et al., 2022a; Ichie et al., 2023; Wang et al., 2021). The reason may be the significant reduction in leaf stomatal morphology (length, width and opening) in *C. multiflorus* during MD (**Figure 3**). This pathway reduces transpiration of *C. multiflorus*. (**Figure 4B**). However, under SD, the leaves had been severely dehydrated, stomata were forced to close, and the number of stomata was significantly reduced (**Figure 3**), leaf Tr was also reduced to a minimum, which is consistent with the findings of (Tardieu and Tuberosa, 2010; Xu et al., 2023). It is shown that *C. multiflorus* controls transpirational water consumption by changing stomatal morphology and density to exchange water with environment.

With the intensification of drought stress, the all-day and daytime stem sap flow of *C. multiflorus* decreased progressively (**Figure 3**). This may due to the fact that leaf water deficit occurs earlier when soil moisture is deficient, in the before noon, the stomatal aperture decreases significantly or partially closes, and the transpiration intensity decreases drastically as a result (Kang et al., 2007). *C. multiflorus* did not show a clear ‘double peak’ under LD and SD, while there was a clear ‘double peak’ under SD (**Figure 3**). It showed that *C. multiflorus* had an obvious ‘lunch break’ phenomenon under SD, and its physiology was severely stressed. It is shown that *C. multiflorus* can survive in soil relative water content above 5 to 10%(SD), but optimal growing conditions are when the soil contains more than 40% (LD) relative water.

During drought stress, the soil water content decreases and the conduits in the xylem of plants are under extreme negative pressure, which results in the failure of xylem function due to embolism of air pockets in the conduits, and water conduction will be limited (Guo et al., 2007). It is noteworthy that with increasing drought stress, *C. multiflorus* had both nocturnal stem rehydrated and nocturnal transpiration during the night, with a gradual increase in nocturnal sap flow in the stem and a gradual decrease in nocturnal transpiration. It was shown that *C. multiflorus* was able to alleviate its own water deficit by increasing nocturnal sap flow and reducing plant capacitance and air cavity formation as a way to enhance drought tolerance and avoid plant hydraulic failure or even death due to drought, which was the same as the study of Klein (Klein et al., 2018; Cochard and Delzon, 2013) and Zeppel (Zeppel et al., 2019).

When *C. multiflorus* was treated with rehydrated after SD, the leaf stomatal morphology and stomatal density indices were able to recover to the level between LD and MD; leaf water potential and RWC were able to recover to levels between MD and SD. In contrast, the variables of stem all-day sap flow, daily sap flow, nightly sap flow and nightly transpiration were not significantly different when compared with the SD. Possibly because when the tree is re-watered, the water potential increases, opening and restoring stomata (Liu et al., 2024), and leaf transpiration resumes again, thereby providing suction for water movement within the tree. However, under SD, xylem tracheal tissue of *C. multiflorus* may experience severe air pocket embolism, which renders the xylem functionally ineffective and limits water conduction (Dai et al., 2017). It was further shown that the stomata of *C. multiflorus* have a strong ability to ‘sense’ and regulate re-watering, and to control transpirational water consumption of *C. multiflorus* by changing the stomatal morphology and density for water exchange with the surrounding environment.

### 4.2 Effect of drought-rehydrated on photosynthetic characteristics of *C. multiflorus*

The results of the present study showed that the more severe the drought stress in *C. multiflorus*, the greater the degree of stomatal closing. This led to a significant decrease in $G_{S}$ and caused a significant limitation of $P_{n}$ , resulting in a significant inhibition of photosynthesis, which was the same as that of the studies conducted by Han (Han et al., 2021), Liu (Liu et al., 2024). Therefore, the self-protection mechanism adopted by *C. multiflorus* under drought stress is one of the main reasons for the limitation of photosynthesis (Wang et al., 2019; Zhang et al., 2019).

Plants can adapt to drought stress by rationally coordinating the relationship between photosynthetic carbon assimilation and water consumption, thereby regulating changes in leaf water use efficiency (WUE) (Mohammad et al., 2023). When under LD (RWC of 25 to 30%), stomata partially closed and both $P_{n}$ and $T_{r}$ began to decline, but the decline in $T_{r}$ was greater than the rate of decline in $P_{n}$ , resulting in an increase in WUE. It indicates that MD can increase the WUE of *C. multiflorus* leaves, which is the same as the findings of Song (Song et al., 2022a).

Drought stress injured both the PSII reaction center and the receptor side of *C. multiflorus* leaves and impeded photosynthetic electron transport (both $PI_{ABS}$ and $F_{\text{v}}/F_{m}$ were decreased). This was mainly due to the fact that drought treatment resulted in the blockage of both electron transfer from the PS II receptor side $Q_{A}$ to the secondary quinone receptor $Q_{B}$ and acceptance of electrons by the plastoquinone PQ pool in *C. multiflorus* ( $V_{j}$ , $V_{i}$ increased). The degree of opening of active PSII reaction centers and the capacity of PQ pools decreased, and the ability of the PSII receptor side of the $Q_{A}$ to transfer electrons decreased ( $\psi_{\text{o}}$ , $\phi E_{O}$ and $\phi R_{O}$ decreased, and $M_{O}$ increased). As well as degradation or inactivation of reaction centers and reduced capture of light energy by antenna pigments ( ABS/RC and $ET_{O}/RC$ decrease). This is the same as the findings of Mohammad (Mohammad et al., 2023) and Wu (Wu et al., 2019). However, when the reaction center of *C. multiflorus* was stressed, it allowed a decrease in trapped light energy ( $TR_{O}/RC$ ) and induced an increase in heat dissipation ( $DI_{O}/RC$ ). This suggests that *C. multiflorus* initiated corresponding defense mechanisms after being subjected to drought stress, defending against excessive accumulation of light energy through reversible inactivation of PSII and reduction of light energy uptake, quantum yield and electron transfer on the one hand, and reducing the accumulation of excess excitation energy by which is the same as the findings of Song (Song et al., 2022b). This is consistent with the research results on the drought resistance mechanism of *Vitis vinifera* L (Shtai et al., 2024). and *Jatropha curcas* (Sapeta et al., 2023).

The rehydrated of *C. multiflorus* after severe drought can photosynthesis and chlorophyll fluorescence to the level of MD. This indicated that the photosynthetic reaction center PSII of *C. multiflorus* had been repaired to a certain extent after the damage, but it had not been restored to the level before drought stress. The photosynthetic reaction center of *C. multiflorus* was still photoinhibited. This is the same as the findings of Wei (Wei et al., 2018).

### 4.3 Coupling of water status and photosynthetic carbon assimilation in *C. multiflorus* and adaptation strategies

The leaf water potential of *C. multiflorus* at SD was -4.97 Mpa, which was 4.44 MPa lower than that of CK, while the $P_{\text{n}}$ , $G_{S}$ , $T_{r}$ and all-day sap flow of *C. multiflorus* at SD were 44.92%, 41.29%, 52.39% and 57.65% of CK, respectively. It may be because the leaf water potential was delayed by 2 days compared with the photosynthetic index measurement, which led to a further reduction of soil moisture and a more serious water stress on *C. multiflorus*. When the stem sap flow and leaf water potential were measured on a different day, the environmental factors (temperature, air humidity, etc.) would affect the sap flow. However, the water status parameters of *C. multiflorus* (except for nocturnal sap flow, the ratio of nocturnal water refilling, and nocturnal transpiration) showed significant or highly significant correlations with photosynthetic characteristics and chlorophyll fluorescence parameters. In particular, stomatal aperture and stomatal conductance of *C. multiflorus* leaves showed significant or highly significant correlations with water status parameters (except for nocturnal sap flow, the ratio of nocturnal water refilling, and the ratio of nocturnal transpiration), photosynthetic characteristics, and chlorophyll fluorescence parameters. This was because stomatal function was capable of controlling plant water status and photosynthesis at the same time (Peláez-Vico et al., 2024). When *C. multiflorus* was subjected to drought stress, the decrease in its hydraulic conductivity, which caused a decrease in the stomatal parameters, further reducing the activity of the photosynthetic apparatus and reducing CO_2_ influx, ultimately leading to a decline in photosynthesis (Han et al., 2022; Bonoso et al., 2020). This suggests a highly coupled relationship between water status parameters and photosynthesis parameters in *C. multiflorus* (Sebastià et al., 2014; Reddy et al., 2019; Limousin et al., 2013).

From the principal component analysis of 25 drought tolerance variables of *C. multiflorus* under different drought degrees and rehydrated status (**Table 1**), it can be seen that the cumulative contribution of principal component I and principal component II reached 96.3513%, and the eigenvalue was >1, which was mainly representative. Although nocturnal sap flow, the ratio of nocturnal water refilling, and nocturnal transpiration of *C. multiflorus* had lower loadings on principal component I, they had higher loadings on principal component II. And with the intensification of drought stress, the proportion of nocturnal sap flow in all-day sap flow and the ratio of nocturnal water refilling tended to increase, reflecting the strategy and ability of *C. multiflorus* to cope with drought changes by compensating for tree sap, which was the same as that in the studies of Li et al (Li et al., 2023), Liu et al (Liu et al., 2023a). This is because the residual effects of water stress are mainly caused by hydraulic dysfunction (Gori et al., 2023), whereas nocturnal sap flow compensates for the internal water deficit of the tree and transports nutrients essential for plant growth and development (Zhang et al., 2022). At the same time, it increased the stem and leaf water potential of the plant before dawn (Fang et al., 2018). In addition, nocturnal sap flow helps to reduce plant xylem embolism and aerial cavity formation, avoiding the phenomenon of plant water failure or even death due to drought (Zeppel et al., 2019). Therefore, nocturnal sap flow cannot be ignored in the study of drought stress in plants.

When *C. multiflorus* is subjected to drought stress, on the one hand, *C. multiflorus* leaves reduce transpiration rate and water loss by changing stomatal morphology and stomatal density, and also maintain their own water balance by increasing the nocturnal sap flow rate and nocturnal water refilling to maintain their physiological and metabolic activities. On the other hand, with the intensification of drought, the $G_{S}$ of *C. multiflorus* leaves showed an overall decreasing trend, and stomatal restriction led to the reduction of photosynthetic performance. But stomata have a strong ability to ‘sense’ and regulate the rehydrated after SD. They exchange water vapour and gas with the surroundings by changing stomatal morphology and stomatal density, thereby control the water consumption of *C. multiflorus.* In addition, when subjected to drought stress, *C. multiflorus* will have activated the appropriate defence mechanisms to defend against excessive accumulation of light energy and promote heat dissipation, protecting the photosynthetic performance of *C. multiflorus* leaves as much as possible. This is the same as drought-avoidant plants such as *Nitraria tangutorum* (Liu and Zheng, 2024), *Genista versicolor* (Albrecht et al., 2024), and deciduous oak (Kaproth et al., 2023). During drought stress, plant morphology and cell metabolism are rearranged (e.g., stomatal closure and leaf rolling, etc.) to avoid or reduce water deficit in plant organs and tissues (Hassan et al., 2023; Perlikowski and Kosmala, 2020). Therefore, to some extent, it can be tentatively assumed that *C. multiflorus* also belongs to drought-avoiding plants.

### 4.4 Stress response of transcriptome factors of *C. multiflorus* under different drought stress and rehydrated conditions

The abundance of transcripts changed significantly in SD, and the number of up-regulated or down-regulated DEGs was relatively large. It was indicated that under SD, the normal physiological function of the leaves of *C. multiflorus* were seriously disturbed, and the gene expression pattern changed dramatically. But *C. multiflorus* had strong tolerance and recovery ability, which was the same as that of *Carya illinoinensis* (Wu et al., 2022), and *Cerasus humilis* (Vastag et al., 2020).

The gene expression of phytosphingosine metabolic process, D-erythro-sphingosine kinase activity, respiratory chain complex III assembly and mitochondrial respiratory chain complex III assembly were significantly down-regulated in SD. It was indicated that SD might lead to the decrease of antioxidant capacity and the inhibition of respiration in *C. multiflorus* (**Figure 9B**), which was the same as the inhibition of antioxidant enzyme system and respiration in *Atractylodes lancea* (Thunb.) DC. under SD (Zhang et al., 2021). The gene expression of Chloroplast thylakoid lumen, tryptophan synthase activity, plastid thylakoid lumen, and tryptophan synthase activity were significantly down-regulated, indicating that SD might cause photosynthesis to be blocked (**Figure 9C**), which was the same as *Xanthoceras sorbifolium* bunge (Liu et al., 2023b).

Although severe drought severely inhibited the respiration, photosynthesis and antioxidant capacity of *C. multiflorus*, it could activate the regulation of stress-activated MAPK cascade and JUN kinase activity. The expression of the gene regulating osmotic pressure and closing stomatal (glyceraldehyde-3-phosphate dehydrogenase, NAD+) was up-regulated, which indicated that *C. multiflorus* would strengthen the drought defense mechanism, promote stomatal closure, reduce transpiration water loss, and vigorously regulate water balance in the body, exhibiting a series of “self-saving” behaviors.

## 5 Conclusion

In conclusion, we systematically investigated the response,coupling mechanisms and molecular mechanisms of water status, photosynthetic characteristics, chloroplast fluorescence parameters and other indicators of *C. multiflorus* in response to drought and rehydrated after drought stress. Our results showed that water status of *C. multiflorus* was highly coupled with photosynthetic characteristics and chloroplast fluorescence parameters during drought stress. *C. multiflorus* could regulate transpiration and photosynthesis by regulating stomatal status and initiating a series of physiological activities through gene regulation. Such as defending against overaccumulation of light energy and promoting heat dissipation, to achieve a balance between hydraulic conductance in response to drought stress. In addition, *C. multiflorus* maintains its own water balance by increasing nocturnal sap flow and nocturnal water refilling to avoid or reduce water deficit in plant organs and tissues. Therefore, the shrub *C. multiflorus* is a drought-tolerant plant.

## Data Availability

The original contributions presented in the study are included in the article/**Supplementary Material**. Further inquiries can be directed to the corresponding author.
